# Increasing prevalence of overweight and obesity in Bangladeshi women of reproductive age: Findings from 2004 to 2014

**DOI:** 10.1371/journal.pone.0181080

**Published:** 2017-07-28

**Authors:** Tuhin Biswas, Md. Jasim Uddin, Abdullah Al Mamun, Sonia Pervin, Sarah P Garnett

**Affiliations:** 1 Health Systems and Population Studies Division, icddr,b, Mohakhali, Dhaka, Bangladesh; 2 Institute for Social Science Research, The University of Queensland, Long Pocket Precinct, Indooroopilly, Queensland, Australia; 3 The Children's Hospital at Westmead Clinical School, University of Sydney, Sydney, Australia; 4 Institute of Endocrinology and Diabetes, The Children's Hospital at Westmead, Locked Bag 4001, Westmead, New South Wales, Australia; McMaster University, CANADA

## Abstract

**Background:**

Overweight and obesity are a particular concern for women of reproductive age. They not only increase the risk of chronic diseases but they are also associated with adverse perinatal, neonatal, infant and child outcomes. The objective of this study was to examine the trend of overweight and obesity among Bangladeshi women of reproductive age between 2004 and 2014.

**Method:**

This is a secondary data analysis of the 2004, 2007, 2011 and 2014 Bangladesh Demographic and Health Surveys (BDHS). We determined the age standardized prevalence of overweight and obesity of women aged 15–49 years, who had their weight and height measured. Overweight and obesity were determined using the Asian specific BMI cut-offs criteria.

**Result:**

The prevalence of overweight increased from 11.4% [95% CI: 10.4to 12.5] in 2004 to 25.2% [95% CI: 24.0 to 26.4] in 2014. The prevalence of obesity increased from 3.5% [95% CI: 3.0to4.2] to 11.2% [95% CI: 10.1to12.5%] over the same period of time. This was seen in all age groups. However, the greatest increase was observed in women aged 35 to 49 years. The highest prevalence of overweight and obesity were observed in those women with the highest education level and wealth, larger family size, living in urban areas and not being in paid employment.

**Conclusion:**

The prevalence of overweight and obesity among women of reproductive age in Bangladesh is high and increasing. We speculate that this has the potential to jeopardize the improvements that have been made in maternal and infant health over the last two decades. Evidence based prevention strategies are required to address this serious public health issue.

## Introduction

Overweight and obesity are an increasing public health problem in both developed and developing countries[1, 2]. In 2014, the World Health Organization (WHO) estimated 39% of adults aged 18 years and over were overweight or obese[3, 4]. The prevalence of overweight and obesity varies widely between countries and tends to increase with income level. Hence, the WHO Region of the Americas has the highest prevalence and the WHO Region for Southeast Asia the lowest[5]. Nevertheless, with increasing economic development in Southeast Asia, increasing rates of overweight and obesity have been reported in most countries including Malaysia, India and Indonesia[6].

The association between overweight and obesity and increased risk of non-communicable diseases has been well described[1, 2].The prevalence of overweight and obesity also varies by sex. In contrast to developed countries, more women in Southeast Asia are overweight and obese compared to men, which in 2013 was estimated to be 28% and 22% for women and men, respectively[1, 4].Overweight and obesity pose an additional concern for women, particularly of reproductive age. It not only affects the woman’s health by increasing her risk of gestational diabetes, type 2 diabetes and cardiovascular disease, it is also associated with adverse perinatal, neonatal, infant and childhood outcomes[7].

As like as other developing country, Bangladesh is experiencing a rapid demographic and epidemiological transition [8–10]which has been associated with increases in overweight and obesity. In 2011, cross-sectional data indicated that the overall prevalence of overweight and obesity combined in women who had been or were married was 18%[11], approximately 1.5 times that reported in women aged 20 to 49 years of age in 2007[12]. However, making a direct comparison in prevalence between years is difficult due to the different age of women in each study; that is ever-married women compared to women age 20–49 years.

It is important to understand the trends in prevalence of overweight and obesity in women of reproductive age for current and future generations and to be able to plan appropriate interventions. To our knowledge this has not been previously described in Bangladeshi women. Hence, the objectives of this study was to determine the trend of overweight and obesity among Bangladeshi women of reproductive age (15 to 49 years) using national representative data between 2004 and 2014 and to examine the socio-demographic determinants of overweight and obesity in the same population.

## Methods

### Participants

This paper analysed secondary data of the Bangladesh Demographic and Health Surveys (BDHS), 2004, 2007, 2011 and 2014. The BDHS is a cross-sectional, national representative survey conducted by collaboration between the National Institute of Population Research and Training (NIPORT), ICF International (USA), and Mitra and Associates. The participants in the BDHS were selected using probability sampling based on a two-stage cluster sample of households, stratified by rural and urban areas in the seven administrative regions of Bangladesh [10]. Urban areas are divided into small administrative units known as mahallas and rural areas are divided into mauzas. Mahallas and mauzas form the primary sampling units in the first stage of sampling and typically include 100–120 households. In the second stage, a sample of 30 households is systematically selected from each primary sampling unit. The detailed protocol and methods were published earlier [13–15]. Data used in this analysis was based on women of reproductive age (ie 15 to 49 years). Women who were pregnant during the survey period or had missing data were excluded from the analysis.

Demographic characteristics, including educational status, involvement in paid work, and region (district) and place (urban or rural) of residence was collected by questionnaire which was administered during a face to face interview. Household wealth index is a composite measure of a household's cumulative living standard. The index is calculated using household's ownership of selected assets, including electricity, televisions and bicycles; materials used for housing construction; types of water access and sanitation facilities; use of health and other services, and in health outcomes. It is determined using principle components analysis. National-level wealth quintiles (from lowest to highest) are obtained by assigning the household score to each de jure household member, ranking each person in the population by his or her score, and then divided the ranking into five quintile, each comprising 20 percent of the population [10, 16].

### Anthropometry

Weight and height were measured at the participant’s home by trained field research staff. Weight was measured twice using a solar-powered scale (UNICEF electronic scale or Uniscale) to the nearest 0.1 kg with light clothing on and without shoes by digital weighing scales placed on a flat surface. Height was measured two times using a standard clinical height scale with participants standing without shoes. The average of the two measurements was used in the analysis.

### Overweight and obesity

Asian specific BMI cut-offs were used to define underweight (<18.5 kg/m^2^), overweight (23.0 to <27.5 kg/m^2^) and obese (≥27.5 kg/m^2^)[17]. The data collection and anthropometric measurements were undertaken by trained field staff in the participant’s home.

### Statistical analysis

We estimated the prevalence of overweight and obesity in the different survey years according to age group. The age-adjusted prevalence of overweight and obesity and 95% confidence intervals (CI) were also calculated. Categorical variables were presented as frequencies and 95% CI. The prevalence of overweight and obesity was presented by age group (15 to 24, 25 to 34 and 35 to49 years) and place of residence. Educational level was stratified into four groups: no education, primary education, secondary education and higher level education. To assess the age differences at different time points, we used logistic regression to estimate the prevalence odds ratio (POR). All analyses were adjusted for sample design (cluster and sample weight) and completed in SPSS (IBM, 21).

### Ethical approval

Ethics approval for the BDHSs was obtained from the Institutional Review Board of the Medical Research Council of Bangladesh. Informed consent was given by the participants.

## Results

The number of participants and the socio-demographic characteristics of the women in 2004, 2007, 2011 and 2014 are presented in Table 1. Between 2004 and 2014 the number of women who had primary and above level education increased from 60.5% to 75.6% and the number of women in paid work increased from 22.6% to 32.4%. Over the same period, the proportion of large families (ie households who had ≥5 residents) declined from 67.2% to 57.6%.

**Table 1:** Demographic characteristics of the participants, 2004 to 2014.

Socio-demographic variables	2004 (n = 10603)	2007 (n = 10127)	2011 (n = 16352)	2014 (n = 16624)
**Age (years)**				
15–24	3342 (31.5)	3041 (29.6)	4670 (28.0)	4478 (26.7)
25–34	3570 (33.6)	3385 (32.9)	5739 (34.4)	6018 (35.9)
35–49	3704 (34.9)	3852 (37.5)	6273 (37.6)	6290 (37.5)
**Educational level**				
No education	4238 (39.5)	3384 (32.9)	4486 (26.8)	4089 (24.4)
Primary	3155 (29.4)	3052 (29.7)	5010 (29.9)	4918 (29.3)
Secondary	2698 (25.1)	3032 (29.5)	5902 (35.2)	6194 (36.9)
Higher	640 (6.0)	807 (7.9)	1365 (8.1)	1585 (9.4)
**Number of members in a household**				
1–2	392 (3.7)	418 (4.1)	710 (4.2)	845 (5.0)
3–4	3132 (29.2)	3201 (31.1)	5781 (34.5)	6280 (37.4)
5+	7207 (67.2)	6659 (64.8)	10272 (61.3)	9661 (57.6)
**Wealth index**				
Poorest	1930 (18.0)	1658 (16.1)	2887 (17.2)	3023 (18.0)
Poorer	1932 (18.0)	1851 (18.0)	3098 (18.5)	3140 (18.7)
Middle	2004 (18.7)	1934 (18.8)	3221 (19.2)	3410 (20.3)
Richer	2138 (19.9)	2071 (20.1)	3570 (21.3)	3559 (21.2)
Richest	2727 (25.4)	2764 (26.9)	3987 (23.8)	3654 (21.8)
**Involved in paid work**				
No	8302 (77.4)	7178 (69.9)	14467 (86.3)	11338 (67.6)
Yes	2428 (22.6)	3096 (30.1)	2296 (13.7)	5443 (32.4)
**Place of residence**				
Urban	3676 (34.3)	3910 (38.0)	5872 (35.0)	5831 (34.7)
Rural	7055 (65.7)	6368 (62.0)	10891 (65.0)	10955 (65.3)
**Region**				
Barisal	1282 (11.0)	1346 (13.1)	1931 (11.5)	2006 (12.0)
Chittagong	1915 (17.8)	1801 (17.5)	2689 (16.0)	2675 (15.9)
Dhaka	2433 (22.7)	2203 (21.4)	2895 (17.3)	2920 (17.4)
Khulna	1612 (15.0)	1615 (15.7)	2537 (15.1)	2470 (14.7)
Rajshahi	2427 (22.6)	1955 (19.0)	2471 (14.7)	2399 (14.3)
Sylhet	1062 (9.9)	1358 (13.2)	2342 (14.0)	2409 (14.4)
Rangpur	-	-	1898 (11.3)	1907 (11.4)

**Fig 1:**
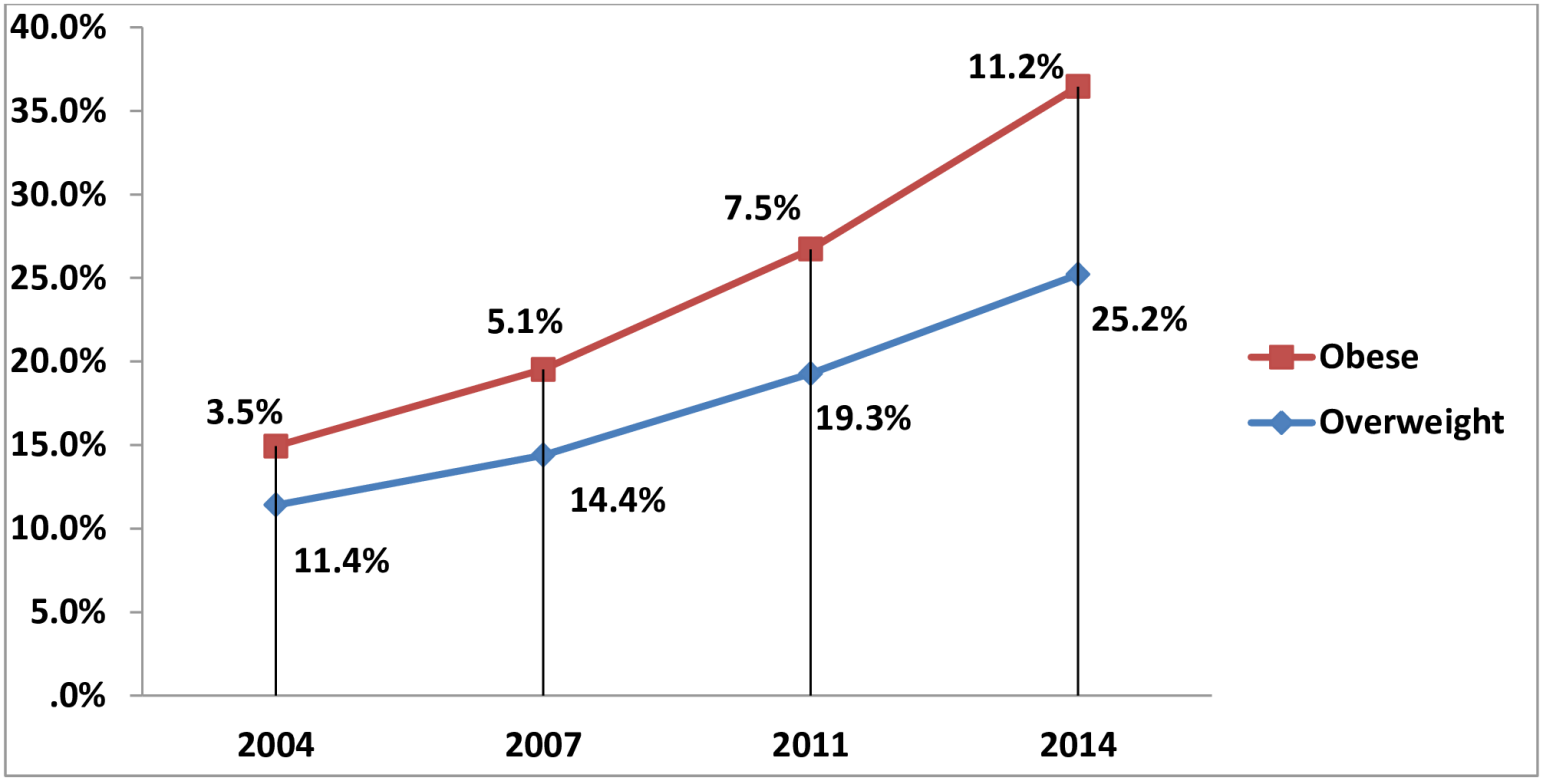
Trend of overweight and obesity 2004 to 2014.

The trends in prevalence in overweight and obesity are shown in “Fig 1”. In 2004, overall 11.4% [95% CI:10.4 to 12.5] of women were overweight, which increased by two and half times to 25.2%, [95% CI: 24.0to 26.4] in 2014. The increase between 2011 and 2014 was 6%. The overall prevalence of obesity increased almost threefold over the same time period, from 3.5% [95% CI: 3.0 to 4.2] in 2004 to 11.2% [95% CI: 10.1 to 12.5%] in 2014.Similar to overweight, the increase in obesity between 2011 and 2014 was four times that seen in the previous four years.

**Fig 2:**
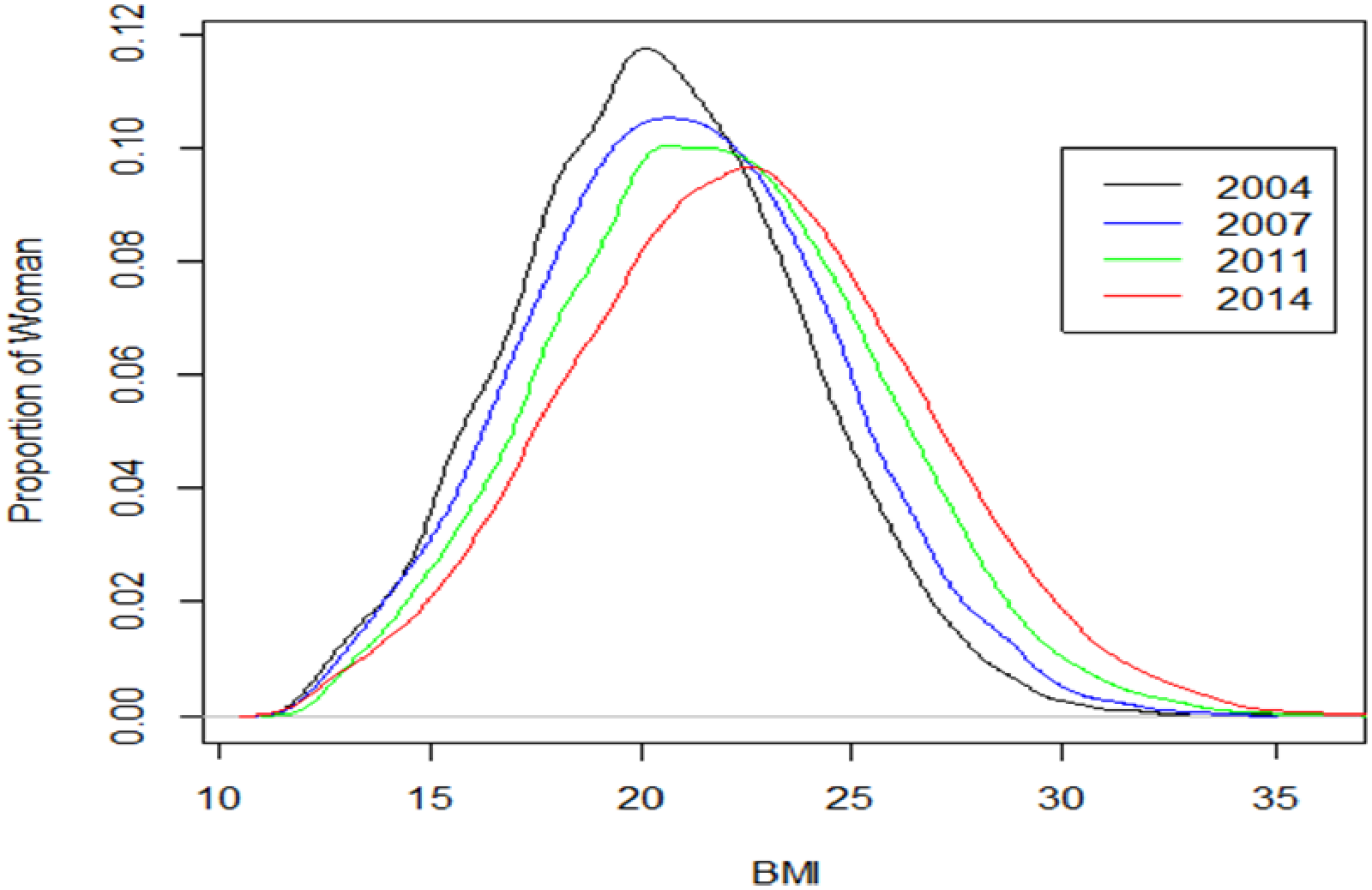
BMI distribution of women from 2004 to 2014 (mean and SD).

Overall BMI distributions of women are shown in “Fig 2”. The mean (SE) BMI increased from 20.31(0.03) in 2004 to 22.31 (0.03) in 2014 (p<0.001). The BMI distribution showed a clear shift to the right, indicating a nutritional transition from underweight to overweight (“Fig 2”).

When the data were stratified by age and place of residence it was noted that in all the four surveys the prevalence of overweight and obesity was less in younger (15 to 24 years) women compared to older women (25 to 49 years) and less in the rural areas compared to the urban areas Figs “3” and “4”. For example, in urban Bangladesh in 2014, 10.8% of 15 to 24 year olds and 23.3% of 35 to 49 year olds were overweight. In the same year, 3.5% of 15 to 24 year olds and 14.0% of 25 to 49 year olds were obese. In comparison in 2014 in rural Bangladesh 5.0% of 15 to 24 year olds and 12.6% of 35 to 49 year olds were overweight and 0.8% of 15 to 24 year olds and 25% of 35 to 49 year olds were obese

**Fig 3:**
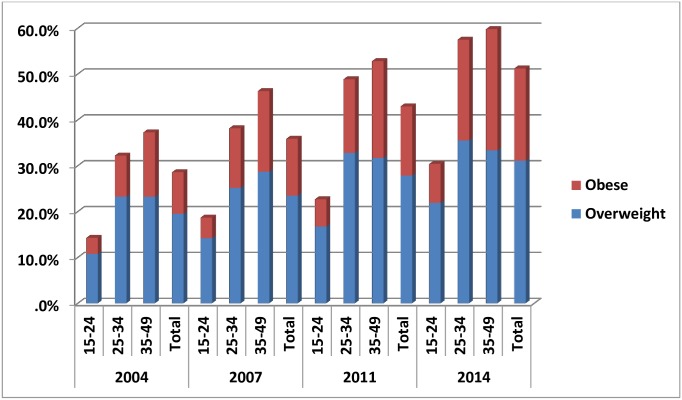
Age specific prevalence of overweight and obesity in urban area.

**Fig 4:**
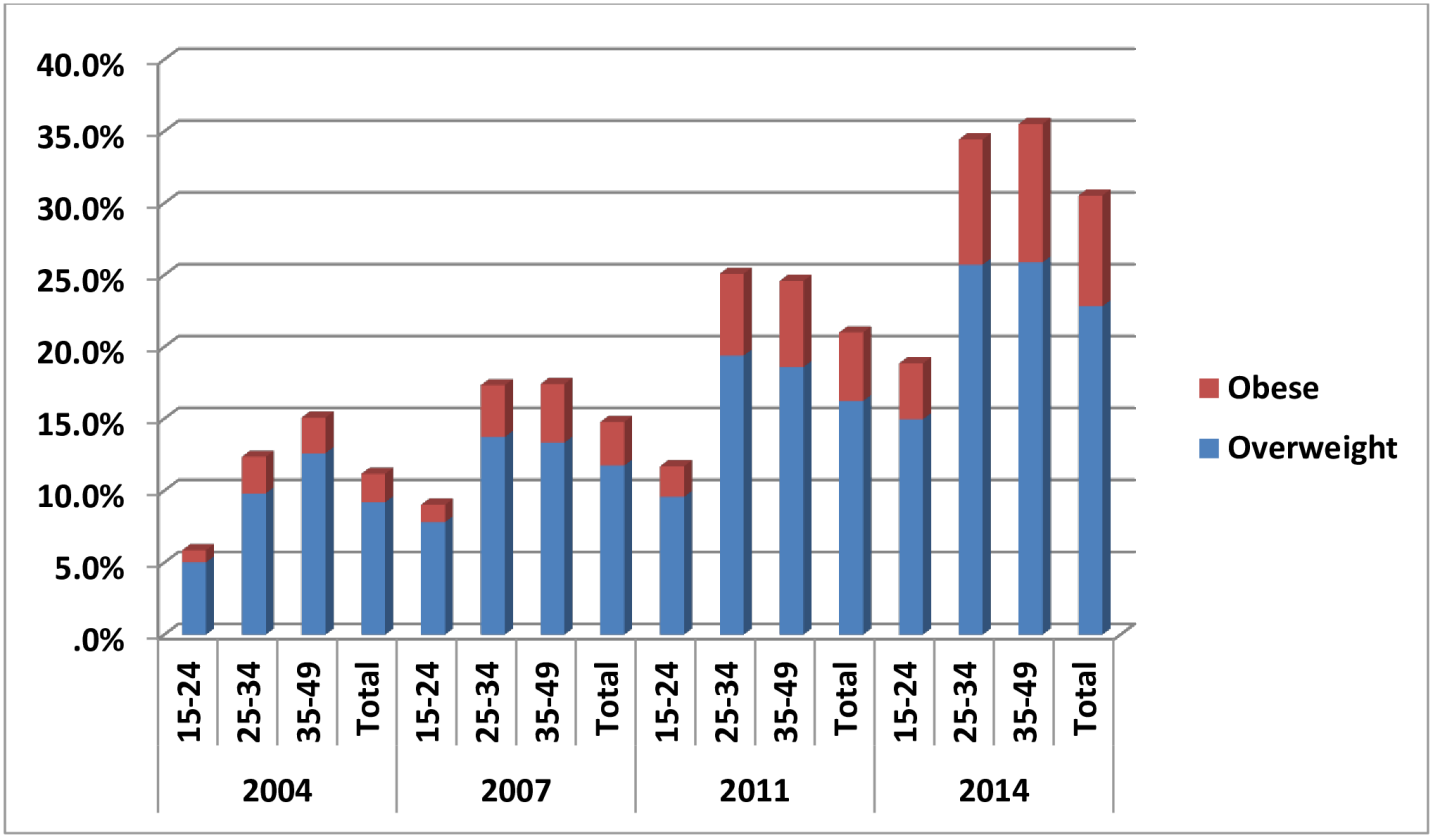
Age specific prevalence of overweight and obesity in rural area.

The PORs for overweight and obesity for women living in urban areas compared with their rural counterparts, stratified by age are shown in Table 2. In 2004, urban women aged 25 to 34 years were four times more likely [POR 2.8:95% CI: 2.2 to 3.5] to be overweight compared to women of the same age livening in rural areas. In 2014, the difference between women living in urban areas compared to rural areas decreased and urban women were twice [OR 1.6:95%CI 1.4 to 1.9] as likely to be overweight. Similarly for obesity, in 2004 urban women aged 25 to 34 years were four times more likely [OR 3.8:95% CI: 2.5 to 5.6] to be obese. In 2014, urban women aged 25 to 34 years were three times more likely [OR 3.0:95% CI: 2.4 to 3.7] to be obese compared to rural women.

**Table 2:** Age specific prevalence odds ratios (POR) with 95% CI associated with overweight and obesity in women living in urban areas compared with women living in rural areas between 2004 and 2014.

Age group	2004			2007			2011			2014		
Overweight	POR	Lower	Upper	POR	Lower	Upper	POR	Lower	Upper	POR	Lower	Upper
15–24	2.3	1.7	3.1	2.0	1.4	2.7	1.9	1.6	2.3	1.6	1.3	2.0
25–34	2.8	2.2	3.5	2.1	1.7	2.6	2.0	1.7	2.4	1.6	1.4	1.9
35–49	2.1	1.6	2.8	2.6	2.1	3.2	2.0	1.7	2.4	1.4	1.2	1.7
**Obesity**												
15–24	4.3	2.3	8.0	3.8	2.2	6.8	2.9	2.0	4.2	2.3	1.6	3.3
25–34	3.8	2.5	5.6	4.0	2.8	5.7	3.2	2.4	4.1	3.0	2.4	3.7
35–49	6.4	4.3	9.6	5.0	3.5	7.2	4.2	3.3	5.4	3.4	2.6	4.4

The highest prevalence of overweight in women of reproductive age between 2004 and 2014 was consistently seen in those with the highest education level (19.0% in 2004 and 26.4% in 2014 for urban women and 4.8% in 2004 and 11.2% in 2014 for rural women) and the richest (15.1% in 2004 and 28.59% in 2014% for urban women and 6.2% in 2004 and 20.8% in 2014 for rural women), Table 3.

**Table 3:** Place of residence specific prevalence of overweight between 2004 to 2014.

Variables	2004		2007		2011		2014	
	Urban	Rural	Urban	Rural	Urban	Rural	Urban	Rural
**Highest educational level**								
No education	11.8 (9.2–14.9)	7.6 (6.5–8.8)	14.4 (11.9–17.3)	9.0 (7.6–10.5)	23 (19.9–26.4)	13.0 (11.6–14.5)	25.0 (21.3–29.1)	18.3 (16.3–20.6)
Primary	14.6 (12.2–17.4)	9.3 (7.7–11.2)	20.6 (17.7–23.8)	11.7 (10–13.6)	23.7 (20.6–27.1)	15.3 (13.7–17.0)	28.0 (24.8–31.5)	21.9 (20.2–23.7)
Secondary	24.7 (21.7–27.9)	10.9 (9.1–12.8)	26.4 (23.4–29.6)	13.6 (11.7–15.8)	29.2 (26.6–31.9)	18.4 (16.9–20.1)	32.0 (29.3–34.9)	25.9 (24.1–27.9)
Higher	38.8 (33.4–44.5)	17.2 (12.6–23)	39.2 (34.7–44.0)	27.4 (21.9–33.6)	38.1 (34.0–42.5)	30.6 (26.2–35.5)	42.1 (37.6–46.9)	30.8 (26.2–35.8)
P value	0.00	0.00	0.00	0.00	0.00	0.00	0.00	0.00
**Number of members in a household**								
1–2	18.5 (12.5–26.7)	11.7 (7.8–17.2)	20.1 (13.6–28.5)	14.3 (10.2–19.8)	28.2 (22.2–35.1)	18.8 (15.0–23.3)	28.4 (23–34.5)	24.7 (20.1–30.0)
3–4	18.3 (15.5–21.5)	9.0 (7.6–10.6)	22.4 (19.4–25.7)	11.8 (10.2–13.6)	28.2 (25.8–30.7)	16.2 (14.7–17.8)	32.1 (29.5–34.9)	24.6 (22.5–26.8)
5+	19.8 (17.0–22.8)	9.0 (8.0–10.2)	24.3 (21.6–27.2)	11.6 (10.2–13.1)	27.6 (25.2–30.1)	16.1 (14.8–17.5)	30.7 (28–33.5)	21.6 (20.1–23.2)
P value	0.68	0.39	0.44	0.49	0.92	0.42	0.42	0.03
**Paid work**								
No	20.2 (17.7–22.9)	9.6 (8.5–10.8)	25.5 (23.4–27.7)	12.7 (11.4–14.2)	28.9 (26.7–31.1)	15.9 (14.7–17.2)	32.2 (30.2–34.2)	23.2 (21.8–24.8)
Yes	16.7 (13.6–20.4)	7.4 (6.0–9.2)	18.9 (15.8–22.5)	9.9 (8.3–11.7)	24.0 (21–27.4)	19.1 (16.6–21.8)	29.0 (25.2–33)	22.1 (20.1–24.1)
P value	0.06	0.03	0.00	0.01	0.01	0.02	0.09	0.27
**Wealth index**								
Poorest	4.5 (2.5–7.9)	4.4 (3.3–5.8)	4.2 (2.0–8.6)	5.4 (4.3–6.8)	10.2 (7–14.6)	7.9 (6.7–9.2)	13.6 (9.3–19.5)	12.7 (10.7–15)
Poorer	3.9 (2.3–6.6)	4.3 (3.2–5.7)	6.3 (4.0–9.8)	8.5 (6.9–10.4)	16.4 (11.8–22.2)	11.4 (10–12.8)	19.2 (14.9–24.5)	18.3 (16.4–20.4)
Middle	13.1 (9.8–17.4)	8.6 (7–10.5)	10.1 (6.7–15)	10.1 (8.5–12.1)	18.5 (14.5–23.4)	16.9 (15.2–18.8)	28.4 (23.2–34.2)	23.8 (21.7–26.0)
Richer	11.5 (8.6–15.3)	14.6 (12.5–16.9)	19 (15.8–22.6)	16.4 (14.3–18.6)	24.2 (21.7–27)	24.1 (22.1–26.2)	27.4 (24.3–30.8)	33.0 (29.6–36.5)
Richest	28.9 (26.3–31.6)	20.4 (17.2–23.9)	31 (28.7–33.3)	29.1 (24.8–33.7)	33.9 (31.3–36.4)	32.5 (29.4–35.7)	37.4 (34.6–40.2)	36.7 (32.5–41.2)
P value	0.00	0.00	0.00	0.00	0.00	0.00	0.00	0.00
**Region**								
Barisal	21.0 (11.9–34.4)	6.9 (5.1–9.3)	19.0 (14.3–25)	9.6 (7–13.1)	30.4 (25.8–35.3)	13.5 (11.2–16.2)	31.9 (29–34.9)	17.5 (14.9–20.3)
Chittagong	19.8 (15.8–24.6)	9.3 (7.5–11.5)	21.5 (17.8–25.8)	11.2 (8.6–14.3)	24.8 (21.1–29.0)	19.3 (16.7–22.1)	30.7 (26.4–35.3)	25.2 (21.8–29.1)
Dhaka	20.2 (16.1–25)	9.8 (7.5–12.7)	24.8 (21.4–28.5)	10.2 (8.3–12.5)	28.8 (25.6–32.3)	13.1 (10.6–16.1)	31.6 (27.7–35.8)	23.1 (20.1–26.4)
Khulna	20.1 (16.3–24.6)	11.1 (8.9–13.8)	23.8 (18.7–29.6)	16.6 (14–19.6)	29.4 (24.3–35.1)	21.9 (19.4–24.5)	34.6 (28.9–40.7)	27.2 (24.7–29.8)
Rajshahi	14.5 (10.8–19.2)	8.4 (6.8–10.4)	24.0 (20.1–28.4)	12 (9.7–14.7)	27.7 (24.6–31.0)	18.9 (16.3–21.7)	31.8 (27.7–36.2)	25.2 (22.3–28.5)
Sylhet	20.1 (12.5–30.7)	7.5 (4.9–11.3)	19.0 (14.8–24)	10.8 (7.2–16)	25.1 (20.2–30.8)	12.8 (10.7–15.1)	28.9 (25.1–33)	19 (16–22.5)
Rangpur	-	-	-	-	26.5 (22.1–31.5)	11.8 (9.6–14.5)	22.4 (17.6–27.9)	16.7 (14.2–19.5)
P value	0.52	0.29	0.44	0.02	0.42	0.00	0.25	0.00

Women who were not in paid employment also tended to have a higher prevalence of overweight. The prevalence of obesity showed a similar pattern to overweight and was highest in those who had the highest education and the richest, Table 4. The largest increase in the prevalence of overweight and obesity was seen in rural women.

**Table 4:** Place of residence specific prevalence of obesity between 2004 to 2014.

Variables	2004		2007		2011		2014	
	Urban	Rural	Urban	Rural	Urban	Rural	Urban	Rural
**Highest educational level**								
No education	3.7 (2.5–5.4)	0.8 (0.5–1.3)	6.1 (4.3–8.6)	1.9 (1.3–2.6)	11.0 (8.6–14)	3.5 (2.8–4.3)	13.5 (10.8–16.8)	5.1 (4.1–6.4)
Primary	7.1 (5.4–9.3)	2.2 (1.6–3)	9.8 (7.7–12.4)	2.8 (1.9–4)	11.2 (8.9–14)	4.1 (3.3–4.9)	15 (12.6–17.7)	7.3 (6.1–8.8)
Secondary	12.8 (9.8–16.6)	3.5 (2.5–4.7)	14.8 (12.1–18)	4.4 (3.3–5.8)	17 (14.6–19.8)	6.2 (5.3–7.3)	24.3 (21.1–27.8)	9.5 (8.2–11)
Higher	19 (14.4–24.5)	4.8 (2.3–9.5)	23.4 (18.6–29.1)	6.7 (4–11)	22.4 (18.6–26.6)	8.6 (6.3–11.8)	26.4 (23.1–30.1)	11.2 (8.5–14.5)
P value	0.00	0.00	0.00	0.00	0.00	0.00	0.00	0.00
**Number of members in a household**								
1–2	5.6 (2.7–11.1)	2.2 (0.9–5.4)	13.5 (7.9–22.1)	1.6 (0.6–4.5)	9.7 (6.2–14.8)	7.5 (5.2–10.6)	14 (9.9–19.4)	9.6 (6.8–13.3)
3–4	9.3 (7.1–12)	1.9 (1.3–3)	13.9 (11.1–17.1)	3.9 (3–5.1)	14.7 (12.4–17.3)	5.3 (4.5–6.3)	20.7 (18.1–23.6)	8.3 (7.2–9.6)
5+	9 (7.2–11.3)	1.9 (1.5–2.4)	11.6 (9.7–13.7)	2.7 (2–3.5)	15.9 (13.6–18.5)	4.3 (3.7–5)	20.2 (17.7–23)	7.2 (6.1–8.4)
P value	0.37	0.96	0.28	0.03	0.07	0.00	0.09	0.10
**Paid work**								
No	10.5 (8.6–12.9)	2.1 (1.6–2.6)	14.9 (12.7–17.4)	3.2 (2.5–4.1)	16.4 (14.2–18.8)	4.8 (4.2–5.4)	22.9 (20.3–25.7)	8.3 (7.1–9.6)
Yes	4.3 (3–6)	1.5 (1–2.4)	6.6 (4.8–9)	2.7 (1.9–4)	10.3 (8.5–12.5)	4.7 (3.5–6.4)	14.1 (11.8–16.8)	6.7 (5.6–7.9)
P value	0.00	0.17	0.00	0.47	0.00	0.96	0.00	0.02
**Wealth index**								
Poorest	0.8 (0.2–3.4)	0.3 (0.1–0.7)	2.5 (0.9–6.8)	0.8 (0.4–1.5)	1.1 (0.4–2.7)	1.7 (1.2–2.3)	4.6 (2.7–7.8)	2.4 (1.8–3.3)
Poorer	1.9 (0.8–4.2)	1 (0.6–1.9)	1.8 (0.8–4.2)	0.9 (0.5–1.7)	2.1 (1–4.3)	2.1 (1.5–2.7)	5.4 (3.4–8.4)	4.8 (3.7–6.2)
Middle	1.7 (0.8–3.5)	1.4 (0.9–2.2)	2.8 (1.5–5.2)	2.9 (2–4)	4.4 (2.9–6.9)	4.1 (3.3–5.1)	10.3 (7.7–13.9)	7.3 (5.9–9)
Richer	4.3 (2.6–7)	3.3 (2.2–4.8)	6 (4.2–8.7)	4.5 (3.2–6.2)	9 (7.2–11.1)	8.1 (6.9–9.4)	15.2 (12.9–17.8)	12 (10.5–13.7)
Richest	15.1 (12.8–17.7)	6.2 (4.3–8.7)	18.2 (15.9–20.7)	10.9 (8.1–14.5)	22.2 (19.8–24.8)	14.8 (12.4–17.5)	28.5 (26.1–31)	20.8 (16.9–25.2)
p value	0.00	0.00	0.00	0.00	0.00	0.00	0.00	0.00
**Region**								
Barisal	10.2 (6.8–14.9)	2.2 (1.4–3.5)	7.3 (4.8–11)	1.8 (1.1–2.9)	13.4 (9.9–17.9)	3.2 (2.2–4.5)	25 (17.9–33.8)	5.5 (4.3–7)
Chittagong	7.6 (4.8–11.8)	2.5 (1.7–3.8)	12.7 (9.5–16.7)	3.5 (2.3–5.5)	13.3 (10.4–16.8)	7.2 (5.6–9.1)	21.4 (17.1–26.5)	9.3 (7.7–11)
Dhaka	10.3 (7.3–14.2)	2 (1.2–3.4)	13.5 (10.5–17.2)	3.5 (2–6)	16.6 (13.3–20.5)	3.6 (2.6–5)	20.3 (16.7–24.5)	7.8 (5.4–11.1)
Khulna	11.5 (7.7–16.8)	1.6 (1–2.7)	8.4 (5.2–13.1)	3.2 (2–5)	15.7 (12.9–18.8)	6.4 (5.3–7.7)	18.8 (16–22.1)	11.5 (9.6–13.7)
Rajshahi	5 (3–8.4)	1.6 (1–2.7)	13.6 (9.8–18.6)	2.7 (1.9–4)	13.4 (10–17.8)	4.6 (3.4–6.1)	17.5 (13.9–21.8)	6.9 (5.4–8.8)
Sylhet	7.9 (5.6–11.1)	2 (1.2–3.6)	8.3 (4.6–14.3)	2.1 (1.1–4)	10.3 (7–15.1)	3.5 (2.5–4.8)	18.8 (14.1–24.7)	5.7 (4.5–7.3)
Rangpur					15.3 (11.4–20.2)	4.2 (2.7–6.4)	13.8 (9.9–18.8)	5.2 (4.1–6.5)
P value	0.10	0.69	0.18	0.59	0.21	0.00	0.34	0.01

## Discussion

We found that the prevalence of overweight and obesity among women of reproductive age in Bangladesh increased by over 2.2 times for overweight, from 11.4% in 2004 to 25.2% in 2014 and 3.2 times for obesity, from 3.5 in 2004 to 11,2% in 2014. Of particular note was that the rate of increase appears to be accelerating overtime potentially indicating that Bangladeshi women are on a trajectory to higher levels. The high and increasing proportion of overweight and obesity that we report threatens the substantial gains that have been made in maternal and infant health in Bangladesh over the last two decades. Overweight and obesity in women during pre-pregnancy or early pregnancy, living in low and middle income countries, has been associated with increased morbidity in the mother, including hypertension, pre-eclampsia and diabetes, more complicated deliveries, post-partum haemorrhage and fetal morbidity and mortality[18]. In India, a neighbouring country with similar rates of overweight and obesity (25% in women >20 years) [6]the burden of overweight and obesity before and/or during pregnancy on health is considered to be greater than under nutrition[19].

The proportion of overweight and obese women in Bangladesh that we report is only slightly lower than that reported in all women living in the South East Region (28% in 2013)[20], despite being one of the poorest countries in the Region. The trend in the increasing prevalence is also consistent with the increasing trend in developing countries and while the prevalence of overweight and obesity tends to be lower in developing countries compared to developed counties, because of the high population of the area, it is estimated where almost 70% of the world’s obese live[6].

Age, higher education and wealth, as well as living in urban areas were factors associated with increased overweight and obesity. Our data indicates that women in successive cohorts are gaining weight at all ages; however the greatest gain was seen in the older age group (35 to 49 years). Previous studies in developing countries have indicated that the highest prevalence of obesity was seen in women in their mid-fifties[21], indicating that the proportion of Bangladeshi women may be higher than what we are reporting in women of reproductive age (15 to 49 years). Another study using the 2011 BDHS data also reported that unemployed urban women at higher risk of being overweight or obese than those women who were involved in manual-labored work[22].

The observation that there was an increased proportion of overweight and obesity in women with a higher socio-economic status in low to middle-income countries, including India, Iran and Vietnam, has also been consistently reported[23–31]. In China, an increase in income has been associated with an increase intake of energy and fat, and consumption of animal and processed foods, all of which are associated with overweight and obesity [32]. We speculate that the association between higher education and wealth and overweight and obesity, in addition to potential dietary differences is due to the nature of the work that the women may undertake; educated women are more likely to engage in jobs that involve less physical activity. A lower prevalence of overweight and obesity were reported in women who are unemployed compared to those who were involved in manual labour [20]. However, in contrast a study in the north-west Iran demonstrated that higher education was negatively correlated with obesity in women, a pattern which is consistent with that observed in high-income countries [28].

This is the first study to examine the trends in the prevalence of overweight and obesity in Bangladeshi women of reproductive age. This is important information for national health policy maker to take initiative to prevent upcoming public health burden. The strength of this study is that it is analysis of large national samples consisting of women living in both urban and rural areas in Bangladesh. However, there are a number of limitations. The surveys are cross-sectional and the analyses presented are associations and causality between nutritional status and determinants cannot be elucidated. In addition, the BDHS data did not include information on dietary habits or physical activity and hence major determinants of nutritional status were not explored.

## Conclusion

We assessed the prevalence of overweight and obesity among Bangladeshi women of reproductive age using a decade of data. The increase in the prevalence of overweight and obesity was especially significant in older, higher educated women with increased wealth. Our study also indicated that the burden of overweight and obesity among women of reproductive age is high and continuing to increase over time. Overweight and obesity in reproductive age are associated with poor reproductive outcomes of women and increased risk of non-communicable diseases and therefore, demands attention from public health program authorities for continuous success in women and child health indicators.
